# Antimicrobial prescribing and infections in long-term care facilities (LTCF): a multilevel analysis of the HALT 2016 study, Ireland, 2017

**DOI:** 10.2807/1560-7917.ES.2018.23.46.1800278

**Published:** 2018-11-15

**Authors:** M Tandan, K Burns, H Murphy, S Hennessy, M Cormican, A Vellinga

**Affiliations:** 1 Discipline of General Practice, School of Medicine, National University of Ireland Galway (NUIG), Galway, Ireland; 2 Health Protection Surveillance Centre (HPSC), Dublin, Ireland; 3 Department of Clinical Microbiology, Royal College of Surgeons in Ireland (RCSI), Dublin, Ireland; 4 Discipline of Bacteriology, School of Medicine, National University of Ireland Galway (NUIG), Galway, Ireland

**Keywords:** antimicrobial, antimicrobial resistance, infections, HALT, multilevel, LTCF, elderly, Ireland, healthcare-associated infections, HAI, antibiotic use, infection control, multidrug resistance, surveillance, modelling

## Abstract

**Background:**

The 2016 point prevalence survey (PPS) of healthcare-associated infections (HAI) and antimicrobial use (AMU) in Irish long-term care facilities (LTCF) (HALT) showed a 9.8% AMU and 4.4% HAI prevalence, based on aggregated data analysis.

**Aim:**

Our aim was to identify institutional and resident risk factors of AMU and HAI.

**Methods:**

HALT 2016 gathered information using institutional and resident questionnaires, for residents who met the surveillance definition of active HAI and/or AMU, limiting analysis to the aggregated institutional level. In January 2017, we requested additional data on age, sex, urinary catheter use and disorientation of current residents from HALT 2016 LTCF and matched to 2016 HALT data.

**Results:**

Of 224 HALT 2016 LTCF, 80 provided additional information on 3,816 residents; prevalence of AMU was 10.6% and HAI was 4.7%. Presence of a coordinating physician (Odds ratio (OR): 0.3; 95% confidence interval (CI): 0.2–0.6), antimicrobial stewardship committee (OR: 0.2; 95%; CI: 0.1–0.6), healthcare assistants (OR: 0.9; 95% CI: 0.9–1.0), antimicrobial consumption feedback (OR: 0.3; 95% CI: 0.1–0.6) and medical care by personal general practitioner (OR: 0.6; 95% CI: 0.7–1.0) were associated with less AMU and feedback on surveillance of infection prevention and control (IPC) practices (OR: 0.6; 95% CI: 0.3–1.0) with less HAI. AMU and HAI varied significantly between LTCF.

**Conclusions:**

Multilevel modelling identified significant inter-facility variation, as well as institutional factors associated with AMU and HAI. An antimicrobial stewardship committee linked with feedback on IPC and prescribing was associated with reduced AMU and HAI.

## Introduction

Residents in long-term care facilities (LTCF) are prone to healthcare-associated infections (HAI) due to co-morbidities with invasive procedures and exposure to indwelling devices [1]. The term LTCF may encompass a diverse range of resident care types, such as general nursing homes, intellectual disability care, psychiatric care, care for physical disability, rehabilitation and mixed-care types [2]. Due to residents’ characteristics, such as co-morbid conditions, physical and functional weaknesses, and living environment, LTCF are a common setting for infections. Infection prevention and control (IPC) is challenging in LTCF because of high antimicrobial use [3,4], with urinary tract infection (UTI), respiratory tract infection (RTI) and skin and soft tissue infection (SSTI) being the most common infections for which antimicrobials are prescribed [5,6]. Prior studies have reported that nearly half of the antimicrobial use (AMU) in LTCF is unnecessary [7,8]. Inappropriate prescribing can be due to the wrong antimicrobial, indication, treatment duration or dosage. Antimicrobials account for 20% of adverse drug events in nursing homes [8]. Long-term AMU, particularly in LTCF, has been linked to *Clostridium difficile* infection (CDI), mucosal candidiasis, pulmonary and liver damage, and increased risk of colorectal adenoma [9,10].

There is substantial variation in AMU and healthcare-associated infections (HAI) between LTCF and between countries [11,12]. In the HALT 2013, the crude AMU prevalence was 4.4% (range: 1% in Hungary to 12.1% in Greece), with a HAI prevalence of 3.4% (range: 0.4% in Croatia to 7.1% in Portugal) [13]. Compared with the EU/EEA overall, the AMU prevalence in Ireland was double (9.8% in 2013 and 2016), even though the HAI prevalence was similar (5.3% in 2013 and 4.4% in 2016) [5,14].

Judicious AMU through active antimicrobial stewardship programmes is essential to slow the emergence of multidrug-resistant organisms (MDRO) [15]. While hospital antimicrobial stewardship programmes reduce the incidence of HAI, MDRO colonisation and CDI, their implementation in LTCF is more challenging [8,16]. The United States (US) Centers for Disease Control and Prevention (CDC) published an antimicrobial stewardship guideline specific to LTCF [17], but no such guidelines exist at the EU level, even though some European countries have specific guidelines for antimicrobial prescribing in LTCF [18]. The decision to prescribe an antimicrobial depends on a number of factors, including clinical situation, advance care plans, utilisation of diagnostic resources, perceived risk by treating physicians, resident demand, the influence of family and nursing staff, and the availability of guidelines [19].

HAI risk factors in LTCF can be related to the individual resident, the environment/institution or the treatments given [20,21]. Resident risk factors include age; length of stay; disability, such as impaired mobility or disorientation; the presence of indwelling devices; multiple comorbidities or chronic skin breaks such as pressure sores [22,23].

The healthcare-associated infections in long-term care facilities (HALT) PPS have been conducted in the EU/EEA on three occasions since 2010, most recently in 2016–17 [5,14,24]. We evaluated the association between institutional and resident factors and AMU and HAI in Ireland, using a combination of HALT 2016 data and additional resident risk-factor data sought retrospectively.

## Methods

### Study design and settings

HALT is coordinated by the European Centre for Disease Prevention and Control (ECDC), according to a standardised protocol, with the aim of evaluating AMU and HAI in LTCF [2]. In Ireland, HALT is a voluntary project coordinated by the Health Protection Surveillance Centre (HPSC), with four national PPS performed to date and increased numbers of participating LTCF each survey (2010: n = 69; 2011: n = 108; 2013: n = 190; 2016: n = 224) [5,14,24,25]. The presented analysis is based on data from the most recent HALT survey conducted in Ireland in May 2016, the full report of which was published in March 2017 [5].

### Study participants

Eligible residents from participating LTCF were included in the study, with demographic information, risk factors, AMU and the presence of active HAI recorded. Residents were considered eligible if they met the surveillance case definition of active HAI and/or were prescribed systemic antimicrobials on the PPS date. HAI was defined using the updated standardised definitions (McGeer criteria [26]) of infection for surveillance in LTCF, published by the Society for Healthcare Epidemiology of America (SHEA) and the US CDC [27].

### Data collection and management

Two paper questionnaires (institutional and resident) were used to collect information [5]. Institutional questionnaires recorded aggregated resident denominator and risk factor data, such as age > 85 years, indwelling device use, etc., along with LTCF bed occupancy, medical care coordination, and IPC and antimicrobial stewardship activities and resources. Resident questionnaires recorded demographic and risk factor information (hospitalisation in the past 3 months, surgery in the past 30 days or the presence of vascular/urethral catheters, incontinence, disorientation or impaired mobility) for residents with active HAI and/or systemic AMU on the PPS date. Completed questionnaires were entered into the HALT software.

**Figure 1:**
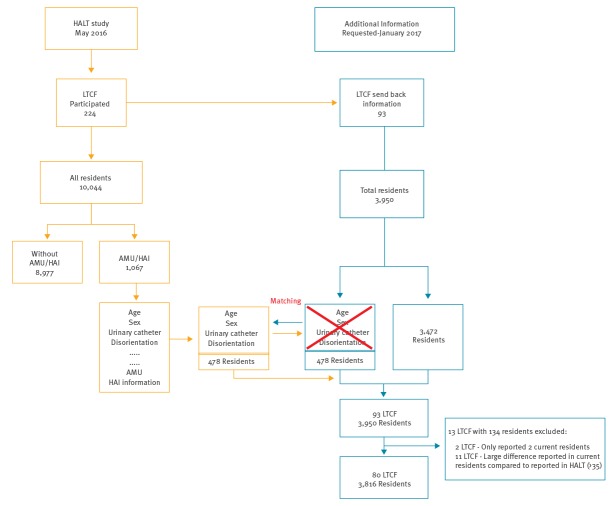
Flow diagram matching HALT 2016 data with additional database, Ireland, 2017

The first analysis was based on Ireland’s HALT 2016 results, looking at aggregated data and the variation between 224 participating LTCF. However, to explore the effects of LTCF characteristics on individuals and to analyse the variation within LTCF or between individuals, more detailed information on all residents is required. Each HALT 2016 participating LTCF was subsequently contacted by the HPSC in January 2017, requesting additional anonymised data on all current residents (age, sex, presence of a urinary catheter and disorientation), with the rationale of limiting the workload associated with additional data collection. The assumption was that each LTCF’s overall resident population would be unlikely to have changed significantly between May 2016 and January 2017. The additional information from each LTCF was matched to the original database, retaining the information of the eligible residents with AMU and/or with HAI to form the ‘additional database’. HALT 2016 residents were matched with those on the additional database by sex and age (closest in age, in some instances), as well as urinary catheter use and disorientation; the case in the additional database was then replaced with the matched case from the original HALT 2016 database (Figure 1).

AMU: antimicrobial use; HAI: healthcare-associated infection; HALT: healthcare-associated infections in long-term care facilities; LTCF: long-term care facility.

### Outcome variables

The outcome variable for the HALT 2016 LTCF was the prevalence of AMU and HAI calculated per 1,000 residents. Mathematically expressed as:

$$Prevalence\;of\;AMU\;=\frac{Total\;number\;of\;residents\;on\;antimicrobials\;on\;the\;day\;of\;survey}{Total\;number\;of\;residents\;in\;LTCF\;on\;the\;day\;of\;survey}\;X\;1,000$$

$$Prevalence\;of\;HAI\;=\frac{Total\;number\;of\;residents\;with\;active\;HAI\;on\;the\;day\;of\;survey}{Total\;number\;of\;residents\;in\;LTCF\;on\;the\;day\;of\;survey}\;X\;1,000$$

The outcome variables for the additional database LTCF were ‘resident with AMU (yes/no)’ and ‘resident with HAI (yes/no)’.

### Predictor variables

In the additional database, age, sex and the presence of a urinary catheter (yes/no) or disorientation (yes/no) were available for each resident. Institutional variables in the multilevel analysis for AMU include those collected as part of antimicrobial stewardship activities; for HAI, these include IPC activities in the LTCF (Table 1).

**Table 1:** Variables available for antimicrobial use and healthcare-associated infection at long-term care facilities, HALT 2016 (n = 224) and additional database (n = 80), Ireland, 2017

Outcome	Variable
Antimicrobial use	• Percentage of residents > 85 years • Percentage of male residents • Percentage of residents with urinary catheter • LTCF type • LTCF size • Number of whole-time equivalent healthcare assistants • Presence of internal coordinating physician for medical care • Physician in charge of medical coordination can consult medical records of residents • Presence of antimicrobial stewardship committee • System to provide feedback to prescribers on antimicrobial consumption • Microbiological sample taken before antimicrobial started • Permission required for prescribing restricted antimicrobials • Presence of at least one antimicrobial prescribing guideline (UTI or RTI or SSTI) • Medical care provided by personal GP or others • Use of a restrictive list of antimicrobials
Healthcare- associated infections	• Percentage of residents > 85 years • Percentage of male residents • Percentage of residents with a urinary catheter • Percentage of residents with pressure sores • Percentage of single rooms • Development of a care protocol • Feedback of surveillance results to staff on IPC practices • Decision on isolation and precautions of residents colonised with resistant microorganisms • Presence of an IPC committee

HALT: healthcare-associated infections in long-term care facilities; IPC: infection prevention and control; LTCF: long-term care facility; RTI: respiratory tract infection; SSTI: skin and soft tissue infection; UTI: urinary tract infection.

Two databases were prepared for analysis: (i) the original HALT 2016 database with institutional data and aggregated resident information and (ii) the additional database with institutional data and individual resident information.

### Statistical analysis

The aggregated analysis of the original HALT 2016 database used a negative binomial regression analysis (a conventional approach) to compare the AMU and HAI prevalence in LTCF. A negative binomial regression was used to model count data when the outcome was overdispersed [28]. This analysis reflects the skewed shape of the outcome variables, such as a high number of zeros or close to zero prevalence. The coefficients were presented as prevalence rate ratios (PRR).

The multi-level logistic regression analysis used the hierarchical structure of the data (residents nested within LTCF) and estimated the chance of a resident having AMU or HAI. The suitability of a multi-level model was checked by introducing LTCF-level variables (random parameters) to the empty model. The empty model (without explaining variables) was compared with models with explaining variables and was considered an improvement if the increase in explained variance is statistically significant (log-likelihood ratio test statistic with p value < 0.05) [29]. Caterpillar plots were generated to compare the variance within and between LTCF. The model-building process used a forward stepwise selection process and individual (resident)-level variables were first introduced followed by group (LTCF)-level variables. Due to high colinearity between explaining variables, each variable was introduced separately and variables with a p value < 0.25 were retained in the model [30].

An adjusted odds ratio (aOR) with 95% confidence interval (CI) for AMU and HAI was calculated for the fixed effects. The Larsen’s median OR (mOR) was calculated for each model to compare the differences in the outcome between LTCF [31,32]. The mOR for each LTCF is the median value of the distribution of the OR when randomly picking two residents from different LTCF, one from a higher risk LTCF and the other from a lower risk one. It confers the theoretical situation of the difference in OR if an identical individual moved from an LTCF with high prevalence of AMU or HAI to one with low prevalence [33,34]. A mOR of 1 signifies no difference between the LTCF in the probability of AMU or the occurrence of HAI [31-33]. For each mOR, a Bayesian credible interval (Crl) was calculated based on the distribution of mOR, comparable to the CI of a fixed-effect OR. The empty model and final model were compared using the Bayesian deviance information criteria (DIC), and a lower DIC value suggests a better model fit [35,36].

Both binomial and multi-level regression analysis was performed in STATA (version 13.0). The Crl for the mOR was calculated in MLwiN (version2.35). A p value of < 0.05 was considered significant. A chi-squared test was used to test the difference between HALT 2016 and additional database information for categorical variables and t-test for numeric variables.

## Results

### Long-term care facilities and residents

In the HALT 2016, 224 LTCF participated. Of those, there were 102 (45.5%) nursing homes (NH), 46 (20.5%) mixed-care facilities and 31 (13.8%) intellectual disability facilities (not shown in Table 2). Of 10,044 residents, 38.2% were male, 38.8% were > 85 years, 6.6% had urinary catheterisation and 3.2% had a pressure sore (Table 2).

**Table 2:** Univariate comparison of general characteristics between the HALT 2016 and additional database, Ireland, 2017

Resident characteristics	HALT 2016 (n = 10,044 residents)		Additional database (n = 3,816 residents)		p value
	N	%	n	%	
Residents with AMU	1,029	10.3	404	10.6	Ns
Residents with HAI	638	6.4	179	4.7	**0.002^b^**
Residents aged > 85 years	3,895	38.8	1,457	38.2	Ns
Male residents	3,836	38.2	1,500	39.3	Ns
Residents with a urinary catheter	661	6.6	287	7.5	Ns
Residents with pressure sores	324	3.2	146	3.8	Ns
**LTCF characteristics**	**n**	**%**	**n**	**%**	**p value**
Single room^a^	5,634	73.6	1,514	75.8	**0.043^b^**
	**Median (SD)**	**Range**	**Median (SD)**	**Range**	**p value**
LTCF size	41.5 (34.3)	5–176	72.0 (45.5)	10–176	Ns
WTE HCA	20.0 (23.5)	0–198	31.1 (43.5)	0–198	Ns

AMU: antimicrobial use; HAI: healthcare-associated infection; HALT: healthcare-associated infections in long-term care facilities; HCA: healthcare assistant; LTCF: long-term care facility; Ns: not significant; SD: standard deviation; WTE: whole time equivalent.

^a^Percentage of single rooms calculated from the total rooms in the LTCF.

^b^Significant at p value <0.05.

Subsequently, in January 2017, 93 LTCF provided additional information. After matching for age, sex, urinary catheter use and disorientation, 13 were excluded from the analysis; two LTCF reported only two current residents each and 11 LTCF reported a large discrepancy in the number of current residents compared with the number reported in HALT 2016 (> 35). Therefore, for 80 LTCF, additional information was reported on 3,816 current residents (Figure 1). Of the 80 LTCF, 404 residents had AMU (10.6%) and 179 had HAI (4.7%) (Table 2). The median age of the residents was 82 and 60.7% were female. Of residents with a urinary catheter, 14.1% had AMU and 17.3% had HAI (not shown in Table 2).

Five of the 224 HALT 2016 LTCF and three of the 80 LTCF reporting additional information had an antimicrobial stewardship committee; 137 and 45 LTCF, respectively, had an IPC committee (Table 3).

**Table 3:** Overview of long-term care facility-level variables, HALT 2016 (n = 224) and additional database (n = 80), Ireland, 2017

Long-term care facility characteristics	HALT 2016 (n = 224)		Additional database (n = 80)					
			AMU			HAI		
	n	%	n	%	p value^a^	n	%	p value^a^
Internal coordinating physician for medical care	60	26.8	23	28.7	Ns	NA	NA	NA
Physician in charge of medical coordination may consult medical records of residents	168	75.0	57	71.3	Ns	NA	NA	NA
Antimicrobial stewardship committee	5	2.2	3	3.7	Ns	NA	NA	NA
Feedback to prescriber on antimicrobial consumption	32	14.3	9	11.3	Ns	NA	NA	NA
Microbiological sample taken before antimicrobials started	43	19.2	16	20.0	Ns	NA	NA	NA
Permission required for prescribing restricted antimicrobials	22	9.8	8	10.0	Ns	NA	NA	NA
Antimicrobial treatment guidelines (at least one: UTI, RTI, SSTI)	116	51.8	36	45.0	Ns	NA	NA	NA
Medical care provided by personal GP only	111	49.5	40	50.0	Ns	NA	NA	NA
Use of a restrictive list of antimicrobial in LTCF	31	13.8	13	16.3	Ns	NA	NA	NA
Development of IPC care protocol	163	72.8	NA	NA	NA	56	70.0	Ns
Feedback of surveillance results to staff on IPC practices	146	65.8	NA	NA	NA	49	61.3	Ns
Decision on isolation and precautions of residents colonised with resistant microorganisms	189	84.4	NA	NA	NA	67	83.7	Ns
IPC committee	137	61.2	NA	NA	NA	45	56.3	Ns

AMU: antimicrobial use; GP: general practitioner; HAI: healthcare-associated infection; HALT: healthcare-associated infections and antimicrobial use in long-term care facilities; IPC: infection prevention and control; LTCF: long-term care facilities; NA: not applicable; Ns: not significant; RTI: respiratory tract infections; SSTI: skin/soft tissue infection; UTI: urinary tract infections.

^a^p values calculated for HALT 2016 vs additional database.

Of the 80 LTCF that provided additional information, 46 (57.5%) had participated in both HALT 2013 and 2016 (not shown in Table 2). The characteristics of the 80 LTCF that provided additional information did not differ significantly from the 224 LTCF participating in HALT 2016, apart from the occurrence of HAI, which was lower in the additional database, while the percentage of single rooms was slightly higher in the additional database (Tables 2 and 3).

### Negative binomial regression analysis

The result of the negative binomial regression analysis showed that LTCF with more catheterised (urinary) residents had higher AMU (by 4%) and HAI (by 10%). None of the other LTCF-related risk factors were found to be associated with AMU or HAI (Table 4).

**Table 4:** Comparison of negative binomial regression and multi-level logistic regression analysis for antimicrobial use and healthcare-associated infections, HALT 2016 and additional database, Ireland, 2017

	Negative binomial regression analysis^a^				Multi-level logistic regression analysis^b^			
	AMU		HAI		AMU		HAI	
	IRR	95% CI	IRR	95% CI	OR	95% CI	OR	95% CI
**Resident-level variables**								
Age	NA	NA	NA	NA	1.01	1.0–1.02	1.0	1.0–1.01
Sex (reference male)	NA	NA	NA	NA	1.1	0.9–1.4	1.0	0.7–1.4
Presence of a urinary catheter	NA	NA	NA	NA	**2.2**	**1.5–3.1^c^**	**2.6**	**1.7–4.1^c^**
**LTCF-level variables**								
% resident > 85 years	1.0	1.0–1.01	1.0	1.0–1.02	NA	NA	NA	NA
% male residents	1.0	1.0–1.02	1.0	1.0–1.01	NA	NA	NA	NA
% resident with a urinary catheter	**1.04**	**1.0–1.05^c^**	**1.1**	**1.0–1.2^c^**	NA	NA	NA	NA
Internal coordinating physician for medical care	0.9	0.6–1.5	NA	NA	**0.3**	**0.2–0.6^c^**	NA	NA
Physician in charge of medical coordination may consult medical records of residents	1.4	0.8–2.6	NA	NA	1.8	1.0–3.5	NA	NA
Antimicrobial stewardship committee	0.7	0.2–1.8	NA	NA	**0.2**	**0.1–0.6^c^**	NA	NA
Feedback to prescriber on antimicrobial consumption	1.4	0.9–2.2	NA	NA	**0.3**	**0.1–0.6^c^**	NA	NA
Microbiological sample taken before antimicrobials started	0.7	0.4–1.0	NA	NA	**2.5**	**1.3–4.6^c^**	NA	NA
Permission required for prescribing restricted antimicrobials	1.1	0.6–1.9	NA	NA	1.4	0.7–3.1	NA	NA
Antimicrobial treatment guideline (at least one: UTI, RTI, SSTI)	0.9	0.7–1.3	NA	NA	0.8	0.5–1.2	NA	NA
Medical care provided by personal GP only	1.3	0.9–1.9	NA	NA	**0.6**	**0.7–1.0^c^**	NA	NA
Use of a restrictive list of antimicrobials in LTCF	1.2	0.9–1.9	NA	NA	1.7	1.0–3.1	NA	NA
LTCF size	1.0	0.9–1.0	NA	NA	1.0	1.0–1.01	NA	NA
WTE HCA	1.0	1.0–1.01	NA	NA	**0.9**	**0.98–1.0^c^**	NA	NA
% single room in LTCF	NA	NA	1.0	0.9–1.0	NA	NA	NA	NA
% residents with pressure sores	NA	NA	1.0	1.0–1.05	NA	NA	NA	NA
Number of single rooms	NA	NA	NA	NA	NA	A	0.9	0.9–1.0
Number of residents with pressure sores	NA	NA	NA	NA	NA	NA	1.0	0.8–1.1
Development of IPC care protocol	NA	NA	0.7	0.4–1.2	NA	NA	1.5	0.8–2.6
Feedback of surveillance results to staff on IPC practices	NA	NA	0.7	0.4–1.2	NA	NA	**0.6**	**0.3–1.0^c^**
Decision on isolation and precautions of residents colonised with resistant microorganisms	NA	NA	1.4	0.7–2.8	NA	NA	1.7	0.8–3.7
IPC committee	NA	NA	0.8	0.5–1.3	NA	NA	1.3	0.8–2.1
**LTCF types (reference. others)**								
Nursing homes	1.1	0.7–1.9	0.7	0.3–1.6	**2.4**	**1.1–5.2^c^**	**2.8**	**1.0–7.5^c^**
Intellectual disability facilities	0.6	0.5–1.7	0.6	0.3–1.3	**6.1**	**2.0–18.4^c^**	1.5	0.4–5.4
Mixed-care facility	1.0	0.6–1.7	0.6	0.3–1.4	2.2	0.9–5.1	2.5	0.9–7.1
**Measures of variation**					**σ^2^**	**SD**	**σ2**	**SD**
Empty Model	NA	NA	NA	NA	0.5	0.2	0.4	0.2
Final Model	NA	NA	NA	NA	0.2	0.1	0.3	0.2
					**mOR**	** 95% CrL**	**mOR**	**95% CrL**
Median OR in Final Model	NA	NA	NA	NA	2.2	1.8–2.8	2.1	1.5–3.1
**Bayesian DIC**								
Empty Model	NA	NA	NA	NA	2,472.5		1,430.4	
Final Model	NA	NA	NA	NA	2,398.2		1,392.6	

σ^2^: variance; CI: confidence interval; CrI: credibile interval; DIC: deviance information criteria; GP: general practitioners; HALT: healthcare-associated infections and antimicrobial use in long-term care facilities; HCA: healthcare assistant; IPC: infection prevention and control; IRR: incidence rate ratio; LTCF: long-term care facility; OR: odds ratio; RTI: respiratory tract infections; SD: standard deviation; SSTI: skin/soft tissue infection; UTI: urinary tract infections; WTE: whole time equivalent.

^a^Binomial regression analysis was performed on all 224 LTCF from HALT 2016.

^b^Multilevel regression analysis on 80 LTCF from additional database, 2017.

^c^Significant p value at < 0.05.

### Multilevel logistic regression analysis

**Figure 2:**
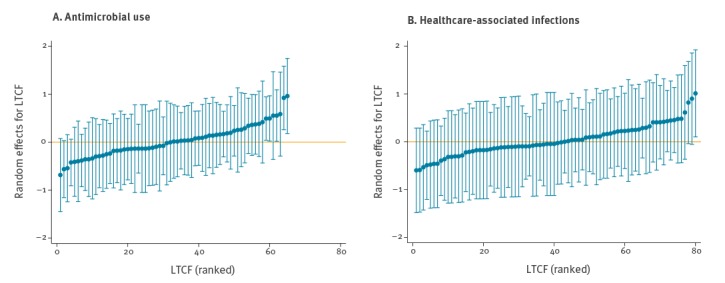
Caterpillar plot showing variance in (A) antimicrobial use and (B) healthcare-associated infections in long-term care facilities^a^, HALT 2016 and additional database, Ireland, 2017

HALT: healthcare-associated infections and antimicrobial use in long-term care facilities; LTCF: long-term care facilities.

^a^After correcting for LTCF characteristics.

The likelihood ratio test, as well as the caterpillar plots, showed substantial variation between LTCF in AMU and HAI (Figures 2A and 2B).

**Figure 3:**
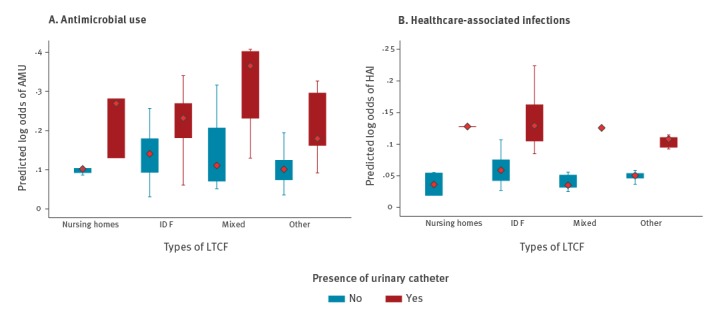
Predicted probabilities of (A) antimicrobial use and (B) healthcare-associated infections by long-term care facility types, HALT 2016 and additional database, Ireland, 2017

HALT: healthcare-associated infections and antimicrobial use in long-term care facilities; LTCF: long-term care facilities.

For both AMU and HAI, significant resident- and LTCF-level variables are presented in the final model (Table 4). AMU was double in residents with a urinary catheter (OR: 2.2; 95% CI: 1.5–3.1) regardless of LTCF type. HAI in residents with a urinary catheter was also double compared with residents without a catheter (OR: 2.6; 95% CI: 1.7–4.1), particularly in residents of intellectual disability facilities, as compared with nursing homes or mixed-care facilities (Figures 3A and 3B).

The presence of an internal coordinating physician for medical care (OR: 0.3; 95% CI: 0.2–0.6), an antimicrobial stewardship committee (OR: 0.2; 95% CI: 0.1–0.6), a system to provide feedback to GP on antimicrobial consumption (OR: 0.3; 95% CI: 0.1–0.6) and medical care provided by personal GP (OR: 0.6; 95% CI: 0.7–1.0) were all significantly associated with reduced prevalence of AMU. An increase in whole-time equivalent (WTE) healthcare assistants (HCA) was associated with reduced AMU prevalence (0.9 for every WTE). Taking a microbiological sample before starting antimicrobials increased the likelihood of AMU by 2.5 (95% CI: 1.3–4.6). The odds of AMU was much higher for nursing home residents (OR: 2.4; 95% CI: 1.1–5.2) and intellectual disability facility residents (OR: 6.0; 95% CI: 2.0–18.4), compared with other LTCF types (Table 4).

Staff feedback on surveillance results of IPC practices was associated with a reduction in HAI (OR: 0.6; 95%: CI 0.3–1.0).

Nursing home residents were nearly three times more likely to have HAI (OR = 2.895% CI: 1.0–7.5) than residents of other LTCF (Table 4).

For both AMU and HAI, large inter-facility differences were observed; the mOR for AMU was 2.2 (95% CrI: 1.8 –2.8) and for HAI was 2.1 (95% CrI: 1.5–3.1), indicating a doubling of the odds for both conditions if an imaginary median resident moved from a lower risk LTCF to a higher risk one (Table 4).

## Discussion

To our knowledge, this is the first multi-level regression analysis of information from the HALT 2016 study. The results showed that with limited additional information, a much more detailed analysis can be performed to reveal associations between institutional, i.e. LTCF, characteristics on AMU and HAI. This approach could therefore be considered to improve antimicrobial stewardship interventions. For future HALT PPS methodology, the collection of age, sex, urinary catheterisation and disorientation status on all residents within each participating LTCF, rather than just residents with AMU and/or HAI, would add to the analysis of data and is recommended.

The aggregated-level HALT 2016 analysis showed urinary catheter use to be the only significant risk factor for both AMU and HAI, while the additional database analysis identified a number of institutional-level variables significantly associated with reduced AMU. These were the presence of an internal coordinating physician, an antimicrobial stewardship committee, feedback to GP on antimicrobial consumption, medical care provided by personal GP and higher numbers of WTE HCA. Staff feedback on surveillance of IPC practices was also directly associated with a reduced HAI prevalence.

The median OR showed high variation between LTCF, with an estimated doubling of the chance of both AMU and HAI for an imaginary median resident if they moved from a low-risk LTCF to a high-risk one. Conversely, the median OR also showed that addressing institutional risk factors could theoretically halve HAI and AMU prevalence.

Formation of a local antimicrobial stewardship committee, linked with feedback on prescribing and/or IPC practices, could positively influence stewardship practices and in turn lead to reduced AMU and HAI. Other institutional changes may require more structural adjustments and resource investments, such as the appointment of internal coordinating physicians or increasing the number of WTE HCA.

### Strength and limitations

The multi-level regression analysis was not specified in advance of the HALT 2016 survey and the additional data collection may have introduced a bias. The additional data on age, sex, urinary catheter use and disorientation may only explain part of the case-mix variability with other factors captured in the ‘unexplained’ variation in the model. However, the comparison of the HALT 2016 database with the additional database did not show any important differences for any of the variables, although only 35% of the participating LTCF responded to the subsequent request for additional information. Most importantly, if larger LTCF contributed more, this would impact analysis and conclusions, as such LTCF may be more likely to have committees or feedback systems. Fortunately, the comparison of the LTCF did not show a bias towards larger or smaller LTCF (data not shown).

The request to LTCF to collect additional data on current residents was pragmatic, taking into consideration staff workload, as a request to retrospectively review data from the HALT 2016 survey was likely to have discouraged the reporting of additional data or limited participation. Therefore, replacing residents with similar characteristics from the HALT 2016 with current residents for whom additional information was collected, thus comparing patients having AMU/HAI with patients who may or may not have AMU/HAI, could have introduced bias. Although a change in outcome is not anticipated by this action, such a bias cannot be checked for nor its direction be anticipated. However, collection of limited additional information on all residents in future HALT studies may show this association to be stronger than was found by our study.

In a PPS, participating residents’ outcome and exposure are measured at the same time, which makes it difficult to derive the direction of the associations found [37]. Finally, the quality of data collected in any PPS depends on good participation and is subject to bias. It is possible that the LTCF that participated in HALT 2016 may have improved awareness of antimicrobial stewardship and HAI prevention, and were therefore more likely to volunteer to participate in a PPS.

### Comparison with existing literature

Antimicrobial stewardship provides standard, evidence-based approaches to encourage judicious AMU [38]. Some perceived barriers in antimicrobial stewardship programmes are physician practice/compliance (69%) and patient/family expectations (15%) [39]. Risk factors identified in relation to clinical practice are the ‘treat first attitude’ and the lack of knowledge regarding effectiveness of antimicrobials, e.g. asymptomatic bacteriuria [40]. In our study, the presence of a coordinating physician, coupled with feedback on antimicrobial consumption, and particularly having an antimicrobial stewardship committee in place, was associated with significantly reduced AMU prevalence. However, only five of 224 LTCF from HALT 2016 and three of 80 LTCF from the additional database reported having an antimicrobial stewardship committee. A nursing home study from Northern Ireland showed appropriate prescribing was associated with regular physician visits [41]. Our study showed the impact of medical care provided by a personal GP in reducing AMU, as GP were considered to be more familiar with the resident’s medical history and conditions over time, which seemed to limit antimicrobial prescribing. Prescribing practices by medical staff other than the personal GP would have been by physicians who were not as familiar with the individual resident’s history [41-44]. Our study supports the appointment of an internal coordinating physician and the maintenance of medical care by personal GP in resident care to support antimicrobial stewardship.

In general, nurses are primarily responsible for resident care in LTCF, supported by HCA who may have more direct resident contact, assisting with personal care, meals and mobility, as required. Some studies suggest that this may result in higher antimicrobial prescribing, specifically for asymptomatic bacteriuria, while other studies suggest that their involvement in prescribing education reduces inappropriate AMU [43,45,46]. Even though nurses and HCA do not prescribe antimicrobials in LTCF in Ireland, our study found no difference in either increased or decreased AMU with a higher or lower number of WTE nurses, but found a modest reduction in AMU with higher WTE HCA in LTCF. This modest reduction may indicate higher involvement of HCA in the direct care of the resident. The HCA role and the nurse to HCA skill mix within LTCF warrants further investigation.

Our study found the practice of taking a microbiological sample before starting antimicrobials to be a key predictor of increased AMU in LTCF, which is similar to a previous study conducted in nursing homes in 2009 [47]. It suggested that the routine of sample taking may be a reminder or justification for prescribing. However, qualitative studies are required to understand such potential association.

In our study, urinary catheterisation was an important resident risk factor associated with higher AMU prevalence. A 2014 study also reported that UTIs were associated with catheter use in both acute care facilities (20%) and LTCF (50%) [48]. A previous study from our group reported an association between AMU and urinary catheterisation, in particular that AMU in catheterised residents was more likely to be prophylactic. According to guidelines, catheterisation is not a sufficient indication for any antimicrobial, either therapeutic or prophylactic [49]. Hence, this is an area where AMU could be improved substantially.

### Conclusion

Collection of some limited additional resident risk factor data after HALT 2016 facilitated multi-level model analysis and thus identification of significant individual and institutional risk factors for AMU and HAI in Irish LTCF, with significant inter-facility variation for both conditions. Our analysis shows the benefit of collecting limited additional information on all residents, which could be considered for inclusion in future HALT PPS. Factors associated with reduced AMU were the presence of a coordinating physician and an antimicrobial stewardship committee, medical care provided by personal GP and antimicrobial consumption feedback to LTCF staff and prescribers. Feedback on IPC practices was associated with lower HAI prevalence.
